# Novel Ultrasound-Guided Sub-Piriformis Injection (SPI) May Produce Effective Analgesia for Both Open and Arthroscopic Hamstring Repair: A Case Report

**DOI:** 10.7759/cureus.74665

**Published:** 2024-11-28

**Authors:** Jonathan P Kline

**Affiliations:** 1 Education, Twin Oaks Anesthesia Services, Wesley Chapel, USA

**Keywords:** open hamstring repair pain management, piriformis, posterior femoral cutaneous, regional block, sciatic

## Abstract

Open or arthroscopic repair of hamstring tear requires both hard and soft, posterior and proximal thigh analgesia. Regional injections to completely relieve this unique pain are not available to the best of our knowledge. We present a novel, single injection, performed under ultrasound guidance, that utilizes the deep piriformis space. This space contains multiple nerves involved in the surgery and can be completely anesthetized with only 10 ml of local anesthetic. We further extended the block for a total of 80 hours through the use of liposomal bupivacaine.

## Introduction

Acute pain management involving open hamstring repair presents unique challenges for anesthesia providers. Both pain management and intraoperative muscle compliance during the procedure are necessary. Traditionally, intravenous (IV) muscle relaxants have been used to provide muscle compliance for repair and posterior sciatic regional blocks used for analgesia. However, in the proximal open hamstring repair, it is possible that neither of these modalities will be effective or complete. In this case study, we present a novel sub-piriformis injection (SPI) regional technique, which when combined with a targeted intramuscular injection, may provide muscle compliance and incisional analgesia. This technique may also include the nearby posterior femoral cutaneous nerve, necessary for proximal incisional analgesia.

The hamstrings are a group of muscles whose function is to flex the knee. This group of muscles is made of four individual muscles known as the biceps femoris long and short head, semimembranosus and semitendinosus. These four muscles receive sensory and motor signals via branches of the common peroneal and tibial nerves, which run through a common sciatic sheath. All four muscles are known to receive blood supply from perforators of the deep femoral artery [1]. Nearly 30 percent of those who suffer from a hamstring injury are likely to re-injure the same muscle within one year of returning to activity [2]. Most hamstring injuries likely occur in high-performance activities requiring maximal speed and direction changes. Hamstring injuries are classified according to a grading system from 1 to 3. Grade 1 injuries are considered mild and traditionally do not require professional consultation. Grades 2 and 3 injuries involving partial and full thickness tears, respectively, are likely to require orthopedic evaluation and surgical intervention [3]. By prevalence, the most frequently injured of the hamstrings is the biceps femoris. This is then followed by the semimembranosus and then the semitendinosus muscles [3-5]. The required regional blockade for such an operation would require denervation of the specific posterior hard and soft tissue regions. The skin and subcutaneous region would be covered primarily by the posterior femoral cutaneous nerve. The posterior femoral cutaneous nerve distribution would contribute almost exclusively to the planned incision [6-8]. The dissection and hamstring muscle repair would be covered primarily by the common peroneal and tibial nerves. These two nerves are encased in a common sheath proximally, deep to the piriformis muscle of the posterior pelvis. The hard tissue, primarily the ischial tuberosity and proximal posterior femur, are innervated by the posterior femoral cutaneous nerve, proximal branches of the tibial and common peroneal and potentially obturator nerves [9].

## Case presentation

A 62-year-old female patient, with American Society of Anesthesiologists (ASA) physical status classification 3, presented for a left-sided arthroscopic vs. open repair of the proximal hamstring. Her past medical history was significant for hypertension, obstructive sleep apnea with continuous positive airway pressure (CPAP) mask use, hyperlipidemia, gastroesophageal reflux disease (GERD), arthritis, chronic low back and sacral pain, hypothyroidism, anemia, anxiety, and depression. Her past surgical history was significant for three open spine procedures (the patient was not knowledgeable regarding procedure type) bilateral total knee replacement, and laparoscopic gastric band placement. She reported a post-anesthesia history of agitation and hallucinations with several surgeries requiring delayed hospital stay and prolonged recovery times. Available pre-operative lab values were all assessed to be within normal ranges. Her allergies included codeine and morphine, which produced itching. Her current medications included Losartan 50 mg PO QD, Tramadol 100 mg PO BID, Celecoxib 200 mg PO BID, Duloxetine 50 mg PO QHS, Pregabalin 50 mg PO TID, Hydroxychloroquine 200 mg PO BID, Alprazolam 0.25 mg PO QHS, Omeprazole 20 mg PO QD, and Progesterone 200 mg PO QHS. 

**Figure 1:**
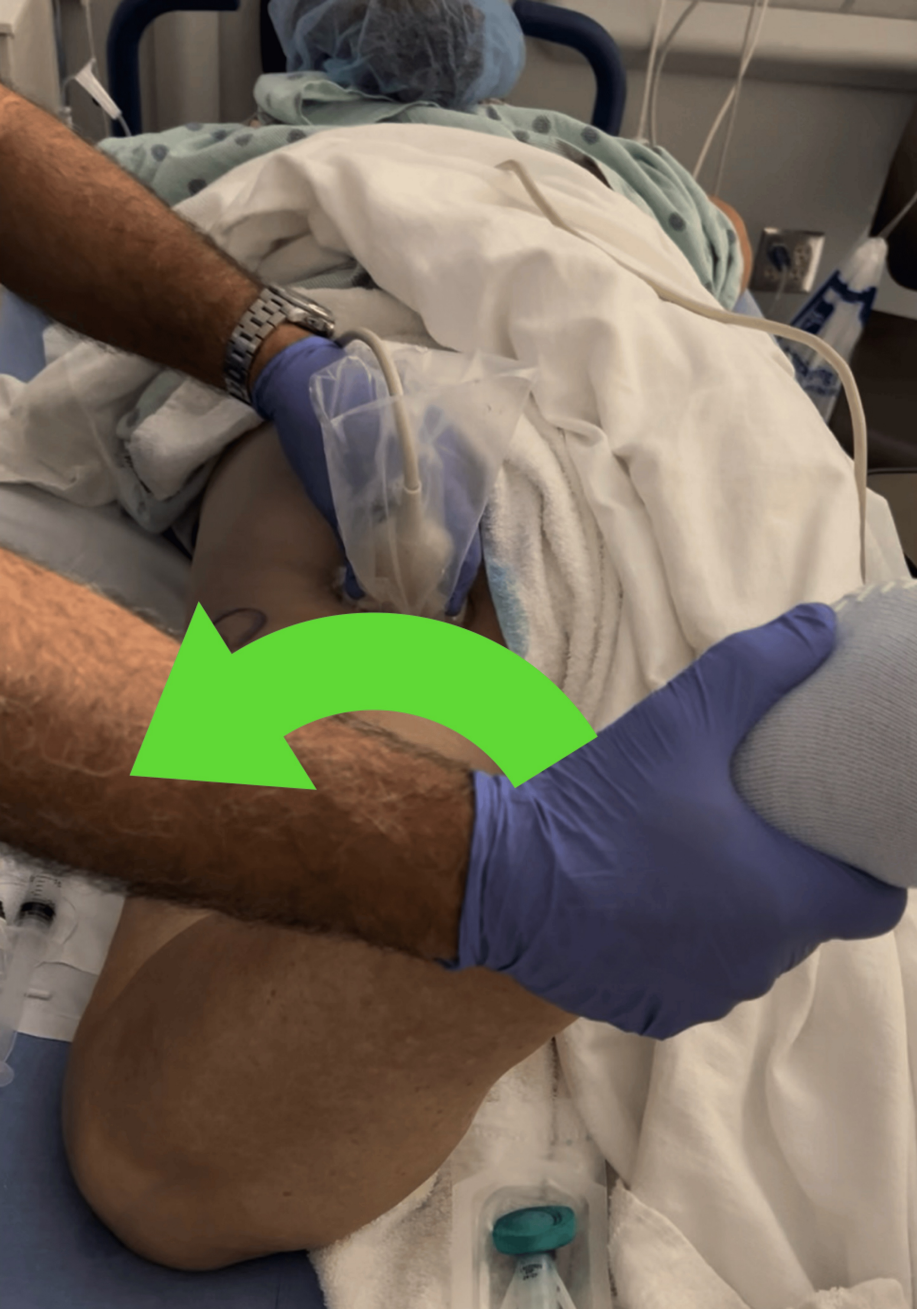
Transverse curved probe positioning, provider ergonomics and internal/external rotation of the femur to verify the piriformis. The green arrow illustrates direction movement while confirming the identity of the piriformis

**Figure 2:**
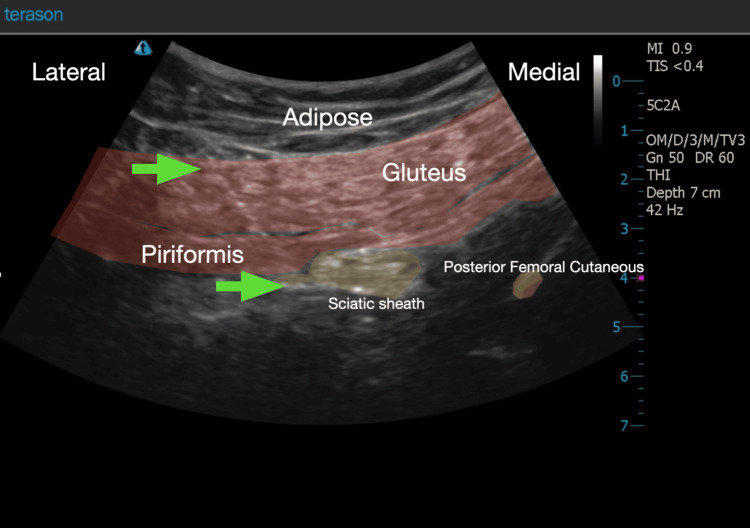
A colored sonogram highlighting the position of sub-piriformis space. This space contains the proximal sciatic sheath, posterior femoral cutaneous and likely the gluteal nerves. The regional block needle can be seen resting below the piriformis muscle adjacent to the sciatic sheath (see green arrows).

The patient was given a pre-operative assessment and informed of the proposed regional technique. The patient demonstrated an understanding of the rationale and necessity of each injection. The first procedure, the novel deep piriformis injection (DPI), aimed to provide analgesia and opioid avoidance. The second procedure, the targeted intramuscular injection, aimed to provide muscle compliance and anti-spasm protection to the hamstring itself. Written and informed consent was obtained and questions were answered to both the patient and family member. A formalized “time-out” was performed and laterality confirmed. While in the pre-operative setting, the patient was placed into a comfortable prone position. ECG, blood pressure and pulse oximetry monitors were applied. A mixture of 10 ml liposomal bupivacaine (Exparel) 13.3% (Pacira Bioscience, Tampa, FL) and 10 ml 0.5% plain bupivacaine was prepared in the same syringe for injection. The patient was given 2 mg Midazolam and privacy ensured. Sterile skin preparation was applied to the skin for the two projected injection sites. Sterile ultrasound acoustic gel was applied to the region just superior to the hamstring gluteal junction, as if a piriformis injection were to be performed. A Terason 3200 Next-Gen (Terason, Burlington, MA) machine was paired with a curvilinear probe. The probe was protected from potential contamination by a probe cover (Safersonic, Highland Park, IL). Sensible ergonomics placed the provider at the patient's left side (operative side), and an ultrasound monitor was positioned on the patient's right side. The curvilinear probe was placed in a transverse orientation, over the inferior gluteal region. The piriformis muscle was identified in a long axis, by its position connecting the greater trochanter and ischial tuberosity. This was visualized dynamically by manually internally and externally rotating the femur. This was performed while the leg was flexed at the knee. This allowed confirmation of the piriformis muscle as it characteristically becomes thickened during internal rotation and thinner during external rotation. This was done while the knee was flexed to provide the desired confirmation of piriformis muscle action (Figure 1). The sub-piriformis space also revealed several distinct features. These included the position of the sciatic sheath, corresponding blood supply and medial region containing the posterior femoral cutaneous nerves (Figure 2). The provider's probe hand was stabilized on the patient and focus placed on image optimization, and prevention of hand drift. A 5 ml of lidocaine 1% was infiltrated into the skin and projected needle track with 25-gauge needle. A 100 mm PAJUNK (Pajunk Medical Systems, Alpharetta, GA) blunt-tipped, echogenic regional block needle was introduced, from lateral to medial. The needle was directed in an in-plane manner, through the piriformis muscle to rest in the sub-piriformal space. Following a negative aspiration, and noting that the patient was free of paresthesias, the sub-piriformis space received 10 ml of the prepared Exparel-Marcaine mixture in a single injection. The patient maintained meaningful contact throughout the procedure.

**Figure 3:**
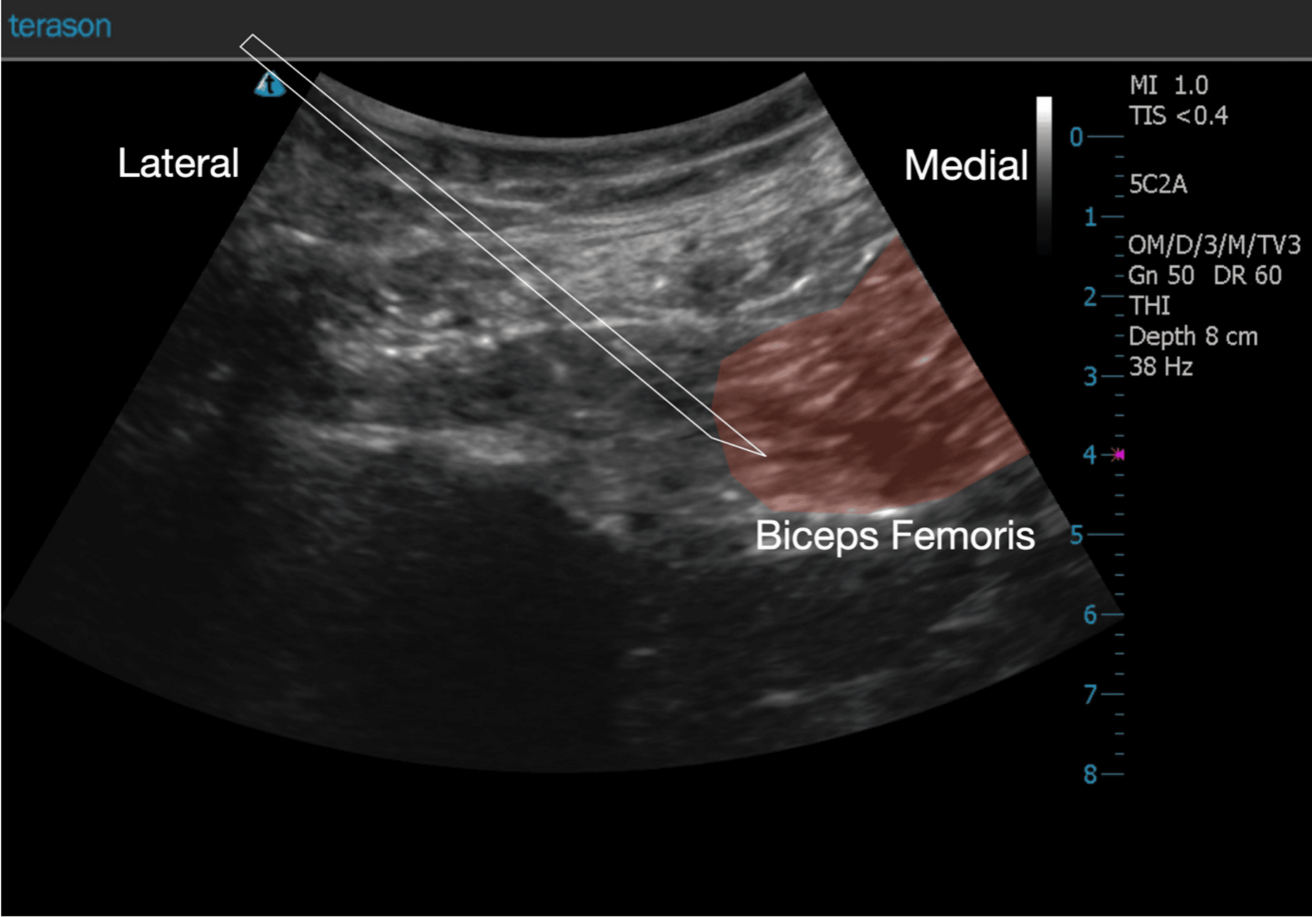
This colored sonogram highlights the short-axis view of the avulsed biceps femoris muscle. This injured muscle is highlighted in red. Note the irregular streaking throughout the muscle. This is relatively inconsistent when compared to non-injured muscle grain appearance. The needle position is outlined to accentuate the trajectory and demonstrate resting position.

The probe was then repositioned inferiorly below the hamstring gluteal junction to reveal the insertion points of the biceps femoris long and short head, semimembranosus and semitendinosus muscles. These were scanned in short axis inferiorly until the tear was located. This appeared as streaking within the muscle itself as well as a hypo-echoic fluid collection around the muscle proximally (Figure 3). The skin and projected needle track were infiltrated using 1% lidocaine via a 25-gauge needle. The same 100-mm blunt-tipped, echogenic regional block needle (PAJUNK) was introduced in plane, from lateral to medial. The needle was directed into the belly of the biceps femoris muscle, corresponding with the hypoechoic fluid collection. The remaining 10 ml of Exparel and Bupivacaine were deposited. This infiltration was performed in a fan-like fashion. The infiltration was similar to the description of the INTRAPEC, another ultrasound-guided intra-muscular injection aimed to create muscle compliance for the surgical field [10].

**Figure 4:**
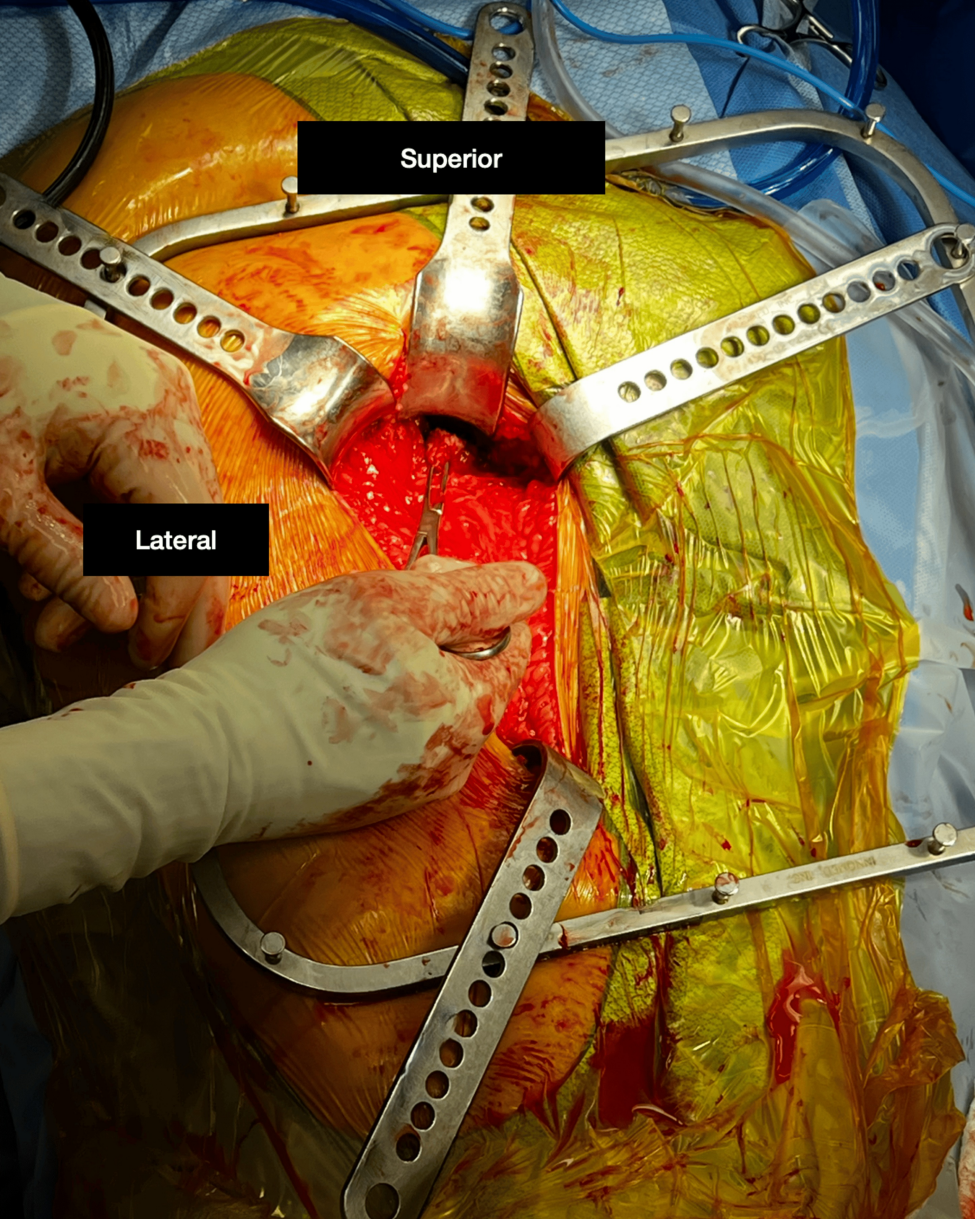
This image shows the actual surgical incision required for ischial tuberosity and hamstring exposure. The surgical clamp is isolating the proximal portion of the biceps femoris tear, just inferior to the ischial tuberosity. The superior designation (12 o’clock on image) represents gluteal region, where the 6 o’clock position represents the inferior portion of the incision.

Shortly after completion of the injections, the patient confirmed pain relief from the injury. The patient also confirmed numbness to the skin of the posterior femoral cutaneous nerve distribution. The patient also demonstrated weakness and tingling into the posterior thigh and sciatic distributions confirming blockade of the tibial and common peroneal regions. The patient was taken to the operating room, monitors applied and pre-oxygenated. The patient received 50 mg IV lidocaine, 200 mg IV propofol and general anesthesia induced with a laryngeal mask allowing for spontaneous ventilation. The eyes were taped, goggles placed and a surgical prone face pillow positioned. The patient was carefully placed into a semi-jackknife, prone position, prepped and draped for an arthroscopic repair of the proximal hamstring muscle. Then 4 mg IV Zofran, 10 mg Decadron, 30 mg Toradol, 2 g Ancef, 1 g Tranexamic acid (TXA) and 1 g Ofirmev were then administered. A surgical malfunction of the equipment forced the surgical team to perform a direct exposure of the retracted hamstring muscle. The incision was extended both superiorly and inferiorly to provide visualization of the retracted hamstring as well as ischial tuberosity (Figure 4). The repair was completed, soft tissue and skin closed and dressing applied. The patient was repositioned supine on the stretcher and emerged from general anesthesia without the use of opioids. The patient was taken to the recovery area. The patient arrived in post-anesthesia care unit (PACU) at 10:41 hrs, was conversant at 10:51 hrs, demonstrated PO (per os) intake at 10:55 hrs and met discharge criteria at 11:11 hrs. She completed ambulation with the assistance of a walker at 11:24 hrs to the restroom and voided. She was subsequently discharged at 11:30 hrs, totaling 49 minutes of recovery room time. Throughout her recovery room stay, she reported no surgical pain and did not require opiates or analgesics of any kind.

The patient was contacted through routine follow-up 48 hours after discharge. At that time, the patient stated that she was still completely free of surgical pain or discomfort relating to the repair. A follow-up phone call was placed six weeks later and the patient was asked to describe block regression. She described block regression in two phases. She claimed the skin sensation returned first at approximately 60 hours post-block. She then stated that the “deep pain“ didn’t begin until about 80 hours' time. She also stated that “by the time the block wore off, I was fine”. She reported no spasm of any kind, for what she described as “the first four days”. She reported that she was more than satisfied with her analgesia and pain control plan following surgery.

## Discussion

The surgical pain generators specific to this case involve proximal branches of the sciatic sheath, posterior femoral cutaneous and direct muscle compliance to the biceps femoris. Traditional sciatic blocks, even those described from a sub-gluteal region, would likely miss the posterior femoral cutaneous distribution, yielding perhaps adequate coverage to the muscles themselves, but not the skin and some of the dissection required for repair. This is particularly relevant to open proximal hamstring procedures. Another relevant point is that despite adequate blockade at the level of the proximal sciatic region, direct contact with surgical electrocautery would still result in further contraction of the sought-after muscle. In this case, the biceps femoris muscle would contract distal to the incision during electrocautery, making surgical efforts to locate and reattach it more difficult. This is a concept potentially lost on the anesthesia provider, which can result in longer dissections, potential blood loss, unnecessary increases in anesthesia exposure and opioid consumption.

Another important consideration for this technique is that, anatomically, the sciatic neural group is not always presented deep to the piriformis. The anatomic relationship of the sciatic sheath to the piriformis may present in a number of different ways. According to Poutoglidou and colleagues, the sciatic nerve group presents deep to the piriformis muscle around 90% of the time. However, the remaining 10% of variations can be vast. This has been described via six different iterations [11]. It is for this reason that regionalists should deliberately visualize the sciatic nerve group, optimize deeper piriformis images and consider use of the peripheral nerve stimulator with this technique.

The analgesia and anti-spasm environment produced by the combination of the regional block and targeted intramuscular injection was likely extended through the use of Exparel vs. standard-acting local anesthetics. It has been demonstrated that Exparel can produce durable analgesia when placed around the tibial and common peroneal nerves. This was formally published by Schwartz and colleagues who demonstrated analgesia lasting over four days following surgery [12].

We recognize that there may be other explanations for the effective analgesia produced following this novel technique. The co-administration of the intravenous (IV) non-steroidal anti-inflammatory drugs (NSAIDs) may have improved the outcome of the proposed regional procedure. However, it should be noted that opioid consumption for the surgery and immediate recovery period was zero. We also recognize that the repair itself may bring some degree of comfort through proper positioning and stabilization of an avulsed muscle attachment. We also recognize that a few similar techniques have been described aiming to block the posterior femoral cutaneous nerve with ultrasound [13,14]. But it should be noted that these techniques deliberately sought to preserve the sciatic motor distribution. This technique sought to include both sciatic components and posterior femoral cutaneous nerves in a single injection. Lastly, it’s likely that other contributory nerves could also be affected by this regional technique. This sub-piriformal space, such as the region described through this technique, may also contain the superior and inferior gluteal nerves. It should also be noted that the anterior space leading into the sacral plexus, deep to the piriformis, can also contain portions of the obturator, pudendal, and piriformis nerves [15]. Incidental blockade of these branches may also contribute to complete analgesia for open or arthroscopic hamstring repair.

## Conclusions

This case presentation demonstrates proof-of-concept that a single, sub-piriformis, ultrasound-guided injection can be effective for successfully blocking the proximal branches of the sciatic sheath and posterior femoral cutaneous nerves. The additional injection into the muscle belly of the biceps femoris likely aided in surgical field muscle compliance needed for repair. This aspect of enhanced muscle compliance may have played a role in more complete analgesia postoperatively. To our knowledge, this is the first reported description of the technique aiming to provide a complete sensory analgesia for proximal hamstring repair with a single injection. Further studies are needed to validate the technique.
